# Chronic kidney disease care delivered by US family medicine and internal medicine trainees: results from an online survey

**DOI:** 10.1186/1741-7015-4-30

**Published:** 2006-12-12

**Authors:** Oliver Lenz, Alessia Fornoni

**Affiliations:** 1 Division of Nephrology and Hypertension, University of Miami Miller School of Medicine, Miami, FL, USA

## Abstract

**Background:**

Complications of chronic kidney disease (CKD) contribute to morbidity and mortality. Consequently, treatment guidelines have been developed to facilitate early detection and treatment. However, given the high prevalence of CKD, many patients with early CKD are seen by non-nephrologists, who need to be aware of CKD complications, screening methods and treatment goals in order to initiate timely therapy and referral.

**Methods:**

We performed a web-based survey to assess perceptions and practice patterns in CKD care among 376 family medicine and internal medicine trainees in the United States. Questions were focused on the identification of CKD risk factors, screening for CKD and associated co-morbidities, as well as management of anemia and secondary hyperparathyroidism in patients with CKD.

**Results:**

Our data show that CKD risk factors are not universally recognized, screening for CKD complications is not generally taken into consideration, and that the management of anemia and secondary hyperparathyroidism poses major diagnostic and therapeutic difficulties for trainees.

**Conclusion:**

Educational efforts are needed to raise awareness of clinical practice guidelines and recommendations for patients with CKD among future practitioners.

## Background

Complications of chronic kidney disease (CKD), such as anemia, metabolic acidosis, nutritional deficits, secondary hyperparathyroidism and hypertension occur well before renal replacement therapy is needed and significantly contribute to morbidity and mortality [1-5]. National treatment guidelines for CKD, and for the management of hypertension, dyslipidemia, bone disease, nutrition, anemia and cardiovascular disease in patients with CKD, have been published [6-12], and dedicated CKD clinics were established based on the conviction that such clinics would help implement Kidney Disease Outcomes Quality Initiative (KDOQI) goals and thus improve outcomes [13,14]. This belief was supported by the observation that patients with already-established advanced CKD who were referred late to a nephrologist had worse outcomes than those referred earlier [15,16], and several groups of researchers [17-20] have postulated that CKD care could be improved by early detection, early referral, and a structured approach to CKD care. An analysis based on the National Health and Nutrition Examination Surveys (NHANES) III and Medicare databases showed that CKD care is suboptimal [21], and recent data suggests that primary care providers may not be familiar will KDOQI guidelines [22]. However, given the vast number of patients with CKD and the limited number of nephrologists [23,24], most patients with early CKD are not seen by a nephrologist. Therefore, this web-based survey was designed to ascertain perceptions and practice patterns regarding CKD care in family medicine and internal medicine trainees.

## Methods

### Study design

The study was approved by the University of Miami's Human Subjects Research Office, protocol number 20060089, under the exempt category. This is a cross-sectional survey of family medicine and internal medicine trainees in the United States, conducted in February and March 2006. Attending physicians' responses served as a control group. The survey was designed to test practice patterns and was based on current KDOQI recommendations.

### Population

Interns, residents, and fellows who were in training in family medicine or internal medicine residency programs in February or March 2006 within the United States were targeted for this survey. A group of attending physicians was included as a control group based on the hypothesis that their responses would provide an insight into the expected knowledge level of trainees at the end of their training.

### Inclusion criteria

Physicians were included in the survey if they identified themselves as physicians practicing family or internal medicine.

### Exclusion criteria

Excluded were respondents other than physicians (such as nurses or students), respondents whose primary specialty was not family or internal medicine, and respondents who did not provide any answer apart from demographics.

### Survey method

The online survey instrument was critically reviewed by four experts in the field, administered to a focus group of five family medicine residents, and modified according to their suggestions. The survey was conducted in an anonymous fashion; no identifying data were retained. The sequence in which choices were presented for questions with multiple answers was randomized for each respondent. All answers except those about age and gender were required to advance in the survey. The survey instrument was posted online on February 1, 2006. Invitations to participate in the survey were distributed by email between February 17, 2006 and March 1, 2006 to 710 internal medicine and family medicine program directors across the United States. It was left up to the program director to decide whether or not to forward the invitation email to residents. A similar email was sent to attending physicians at the PI's center (University of Miami Miller School of Medicine, Miami, FL, USA) on March 3, 2006. The survey instrument was open for responses from February 17, 2006 to March 16, 2006. No incentive was offered for completion of the survey. A copy of the survey instrument is enclosed in  Additional file 1. We also surveyed ten nephrology fellows and attendings to have an internal "positive control", and the data they provided are enclosed in  Additional file 2.

### Statistical analysis

Data are presented as descriptive statistics derived from cross-tabulation. Proportions were calculated based on the total number of respondents for each question and compared using Fisher's exact test assuming a normal distribution. Questions about risk factors were dichotomized into two groups, with those responding that the individual risk factor highly increases risk coded as yes and those responding that it does not or slightly increases risk coded as no. All analyses were carried out using SPSS 14.0 for Windows (SPSS Inc., Chicago, IL, USA). Area proportional Venn diagrams were constructed using the web-based application developed by S. Chow and P. Rodgers [25,26].

## Results

### Study population

A total of 463 responses were received. A response rate could not be calculated given that the target population was not queried directly but through program directors. Fifty-one datasets were blank and were discarded; the remaining 412 data sets were retained for analysis. One hundred and sixty-two responses were received from family medicine trainees, 214 from internal medicine trainees, and 36 from attending physicians. Of the latter group, six were family practitioners, and 30 were internists. Among the internists there were one endocrinologist, one gerontologist, one oncologist, three infectious disease specialists, six cardiologists, and 18 general internists. Both trainees and attending physicians reported seeing a median of six patients with chronic kidney disease per week.

### Recognition of risk factors for chronic kidney disease and assessment of kidney function

**Figure 1:**
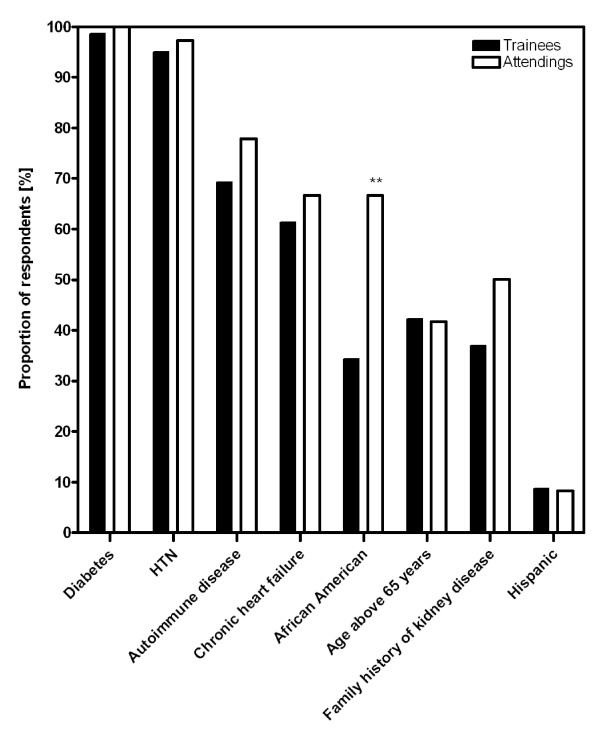
**Identification of risk factors for CKD**. Shown is the proportion of respondents who stated that the shown conditions "highly" increase the risk to develop chronic kidney disease. HTN: hypertension. **p < 0.005 (all trainees versus attendings).

Participants were asked to rank a set of factors as increasing the risk for CKD "minimally", "moderately", or "highly". Answers were dichotomized, with "highly" being categorized as "yes" and the other two options as "no". The results are shown in Figure 1. The responses by family medicine and internal medicine trainees were not significantly different, and thus their responses were pooled. Compared to trainees, a significantly higher proportion of attendings identified African-American race as a high risk to develop CKD (p < 0.005).

**Figure 2:**
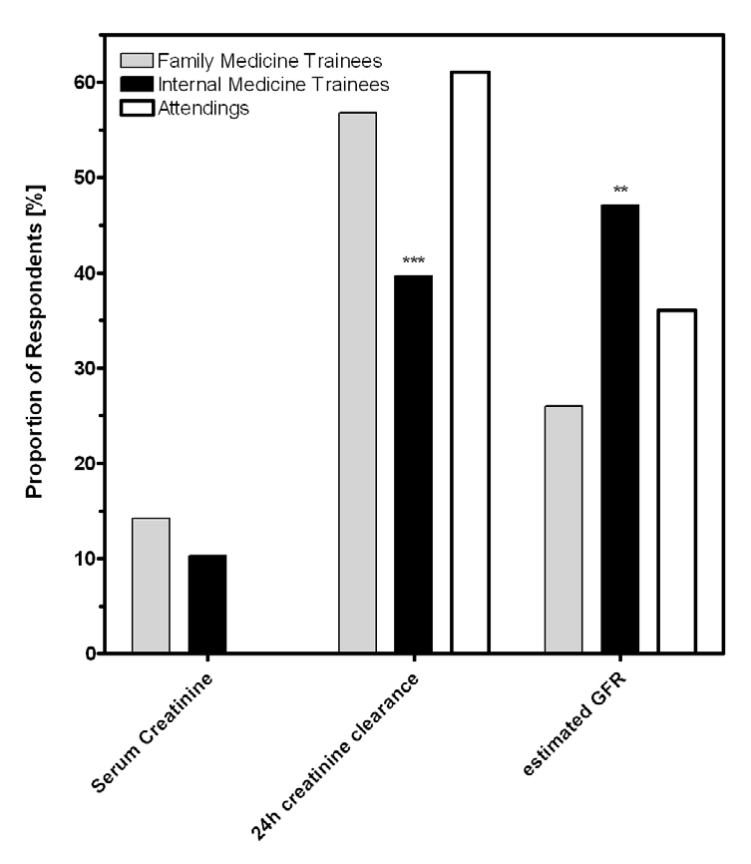
**Evaluating kidney function**. Shown is the proportion of respondents who identified serum creatinine, measured creatinine clearance, or estimated glomerular filtration rate (GFR), respectively, as the best method to evaluate kidney function. **p < 0.005; ***p < 0.0005 (family medicine versus internal medicine trainees).

Participants were asked to identify the best method to estimated kidney function (Figure 2). Timed urine collection for creatinine was the most common choice, followed by estimated glomerular filtration rate (eGFR) using a mathematical formula. Compared to family medicine residents, a significantly higher proportion of internal medicine trainees, but not attending physicians, preferred eGFR over measured creatinine clearance (p < 0.0001).

### Blood pressure goal and therapy choices

**Figure 3:**
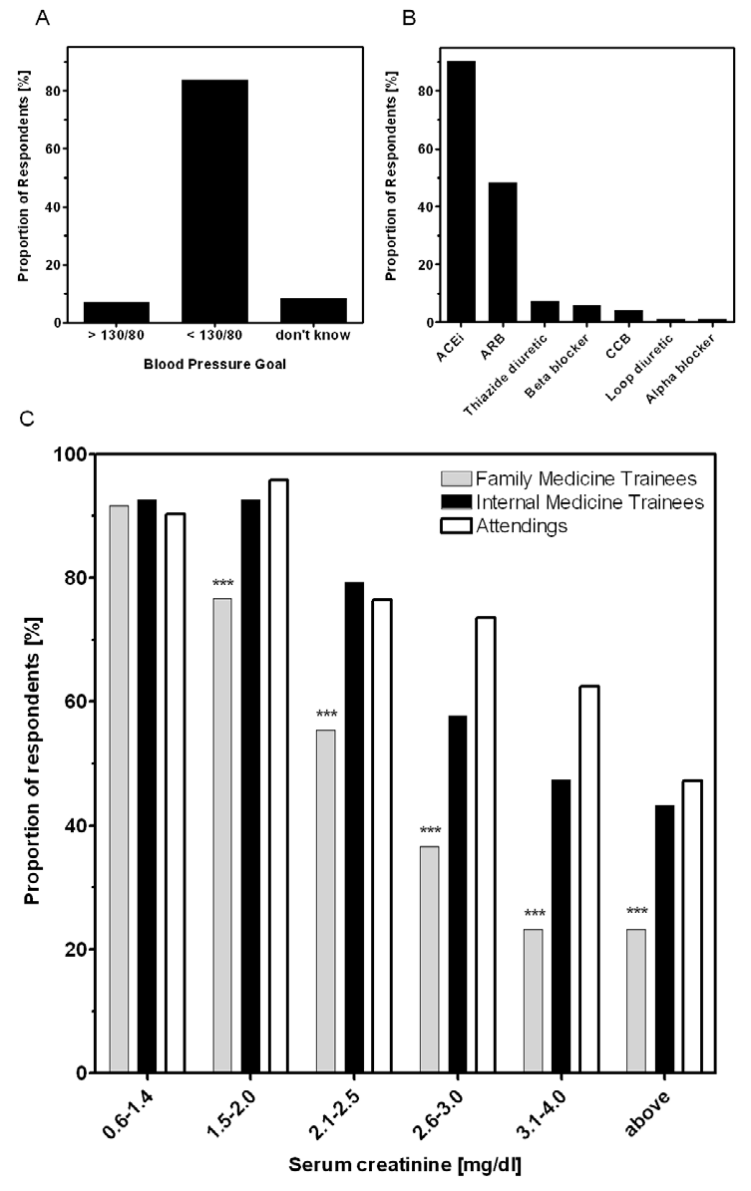
**Blood pressure management in CKD patients**. Panel A shows the blood pressure goals chosen for patients with stage 3 CKD. Panel B shows the classes of antihypertensive agents identified as first-line therapy to slow the progression of renal failure in patients with mild to moderate renal impairment. For panels A and B, data were pooled from all respondents as no differences were found between trainees' or attendings' responses. Panel C shows the proportion of respondents who would add an angiotensin converting enzyme inhibitor to the regimen of a hypertensive patient with microalbuminuria currently treated with a thiazide diuretic, as a function of the patient's serum creatinine. ***p < 0.005 for family medicine versus internal medicine trainees and p < 0.05 for family medicine trainees versus attendings. p > 0.05 for internal medicine trainees versus attendings.

The vast majority of respondents chose a blood pressure goal of less than 130/80 mmHg for a hypothetical patient with CKD and a creatinine clearance of 40 ml/min (Figure 3A). The question posed was: "Which class of antihypertensive medication was considered a first-line agent to slow the progression of kidney disease in a patient with mild to moderate CKD?" (Figure 3B). The responses from family medicine trainees, internal medicine trainees, and attendings were not statistically different for the data shown in Figures 3A,B, and were therefore pooled. Angiotensin-converting enzyme inhibitors (ACEi) or angiotensin receptor blockers (ARB) were the first-line medications chosen by 90% and 48% of respondents, respectively, and only 6% chose neither of the two. Respondents were then given a case scenario of a 37-year-old man with hypertension, microalbuminuria, and a blood pressure of 145/95 mmHg, who is only treated with a thiazide diuretic. Asked if they would add an ACEi to this patient's regimen, a decreasing number of respondents opted to do so as the patient's serum creatinine increased (Figure 3C). Family medicine trainees were significantly less likely to choose an ACEi once the serum creatinine was greater than 1.4 mg/dl (P < 0.0001). There was no difference in the response whether the scenario was given for an African-American or a Non-Hispanic Caucasian man (data not shown).

### Complications of CKD

**Figure 4:**
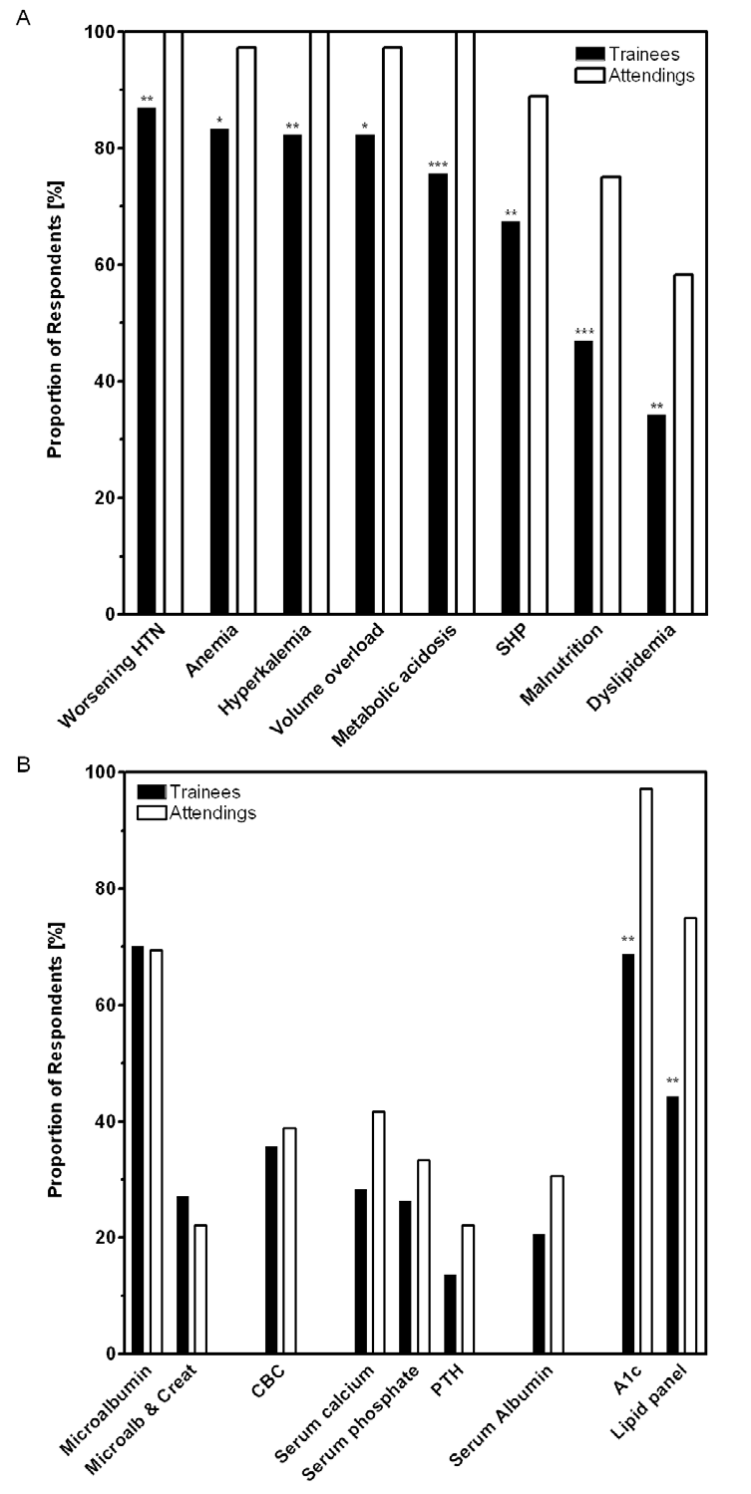
**Complications of CKD**. Panel A shows the proportion of respondents who identified the listed conditions as co-morbidities of CKD (*p < 0.05; **p < 0.01; ***p < 0.005 for trainees versus attendings). Panel B shows the proportion of respondents who would order the listed laboratory data in a hypothetical patient (55 year-old African American woman with diabetes, hypertension, and a serum creatinine of 2 mg/dl). Data for family medicine and internal medicine were pooled because the two groups were not different from each other. **p < 0.01 for trainees versus attendings.

When asked what conditions were complications of CKD, more than 80% of all respondents recognized worsening hypertension, anemia, hyperkalemia, and volume overload. A smaller proportion of family medicine residents than internal medicine trainees chose metabolic acidosis (76% vs 85%, P < 0.05) and secondary hyperparathyroidism (SHP; 61% vs 86%, P < 0.0001). A smaller proportion of trainees identified malnutrition (47% and 54%) and dyslipidemia (32% and 42%) as CKD complications. The proportion of attendings was higher than the proportion of trainee for any of the choices (Figure 4A). A case scenario of a 55-year-old African-American woman with hypertension, type 2 diabetes, and a last known serum creatinine of 2 mg/dl, was given. The question was posed: "Which of the following laboratory tests would you order in a patient like this?" in order to determine what complications trainees were likely to screen for (Figure 4B). Seventy percent of all respondents requested microalbumin in a random urine sample, but only 19%, 37%, and 23% of family medicine trainees, internal medicine trainees and attendings, respectively, concomitantly requested a urine creatinine. Although more than 80% of trainees identified anemia as a complication of CKD, only 36% requested a complete blood count in this hypothetical patient with stage 3 CKD. Screening for SHP and nutrition reflected the low rates of recognition shown in the prior question (Figure 4A). Moreover, when screening for SHP, 10%, 21% and 20% of family medicine trainees, internal medicine trainees, and attendings, respectively, only requested serum calcium and serum phosphate, and only 6%, 19%, and 23%, respectively, requested a parathyroid hormone (p = 0.002 for family versus internal medicine trainees), either alone (1%, 5%, 10%) or together with calcium and phosphate (5%, 14%, 13%). All except two attendings (94%) indicated that they would request A1c, while only 74% of trainees did so (P < 0.0005). A similar discrepancy between attendings and trainees was seen for the lipid panel (75% vs 48%, p < 0.005).

### Anemia and secondary hyperparathyroidism

**Figure 5:**
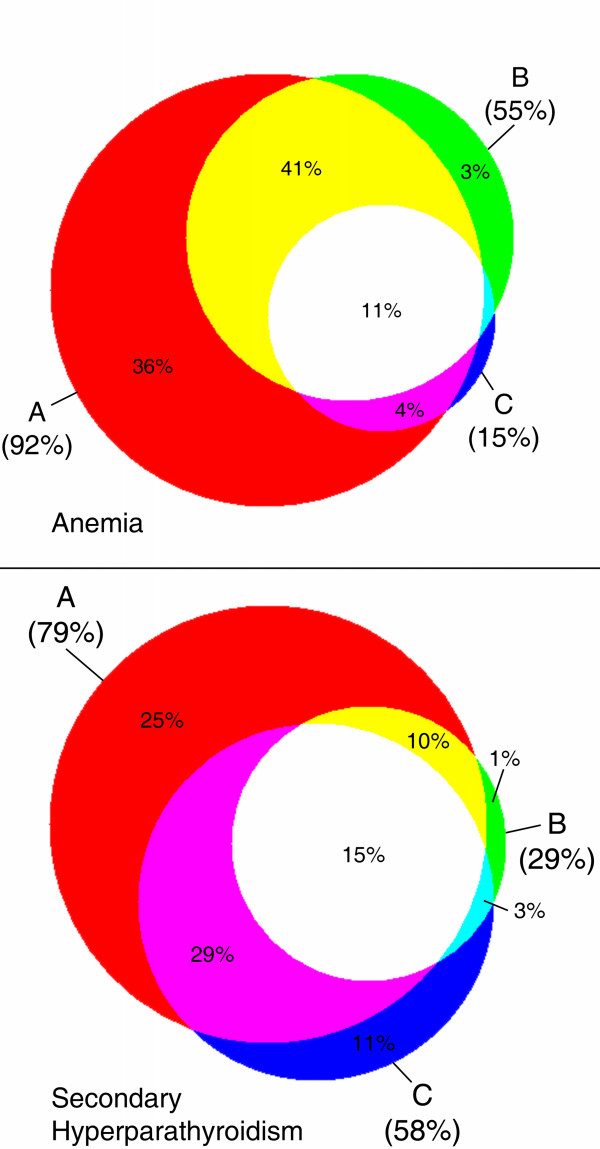
**Management of anemia and secondary hyperparathyroidism**. The top panel shows the proportion of respondents who indicated that anemia is a complication of CKD (A, 92%), that they screened starting in stage 3 CKD or earlier (B, 55%), and that they intervened at a hemoglobin concentration of less than 11 g/dl (C, 15%). Overlapping areas correspond to the proportion of participants whose responses fulfilled two adjacent or all three criteria (white, 11%); the two blue areas correspond to less than 1% each. The bottom panel shows the proportion of respondents who indicated that secondary hyperparathyroidism is a complication of CKD (A, 79%), that they screened starting in stage 3 CKD or earlier (B, 29%), and that they intervened at an intact PTH concentration of more than 70 pg/ml (C, 58%). Overlapping areas correspond to the proportion of participants whose responses fulfilled two adjacent or all three criteria (white, 15%).

Three additional questions concerning screening and management of anemia and SHP were posed: (1) is it a complication of CKD;(2) at what stage of CKD do you screen for it; and (3) at what level of hemoglobin (Hgb) or parathyroid hormone (PTH), respectively, do you start therapy or ask for a consultation when faced with a patient whose creatinine clearance is 40 ml/min. Only respondents who answered all three questions were included in the analysis (N = 312). Data from all respondents, family and internal medicine trainees and attendings, were pooled for this analysis and represented as area proportional Venn diagrams (Figure 5). In each diagram, area A represents the proportion of respondents who identified the condition as a complication of CKD, area B indicates the proportion of respondents screening for the condition in stage 3 CKD or earlier, and area C shows the proportion of respondents who started therapy or asked for a consult at a Hgb < 11 g/dl or a PTH >70 ng/ml, respectively. Among all respondents, 92% identified anemia as a complication of CKD, 55% indicated that they would screen at stage 3 CKD or earlier, and 15% would intervene at a hemoglobin of less than 11 g/dl in a patient with a creatinine clearance of 40 ml/min, resulting in only 11% of respondents who correctly integrated all three parameters. Similar results were seen for SHP management, with only 15% of respondents identifying SHP as a CKD complication, screening at CKD stage 3 or earlier, and intervening once intact PTH surpasses 70 ng/ml in a patient with a creatinine clearance of 40 ml/min.

## Discussion

Delayed referral of CKD patients to a nephrologist has been identified as an important predictor of poor outcomes [15,16]. However, in order to initiate timely referral of CKD patients, primary providers need to be aware of risk factors for CKD and co-morbidities associated with CKD, as well as clinical practice guidelines describing optimal CKD care, such as KDOQI guidelines, the recently-published recommendations from the American Diabetes Association (ADA) [27], or the clinical practice guidelines developed by the Renal Physicians Association [28], the latter being published as an executive summary with clear "hands-on" guidance as to the management of patients with an estimated GFR of less than 30 ml/min/1.73 m^2^. Several educational efforts are currently under way to enhance awareness of CKD [29,30]. However, the data presented in this report show that both family medicine and internal medicine trainees have important knowledge gaps when it comes to CKD care.

Similar to data recently published by Lea et al [22], almost all physicians identified diabetes and hypertension as strong risk factors, while minority status or family history did not receive the same recognition. This may be due to the absence of a unifying model predicting CKD risk in the general population [31]. Some risk factors, such as diabetes and hypertension, have clearly been established [32]. However, attempts to quantitate the contribution of others, such as race, ethnicity, and socio-economic status, has proven to be more challenging because of the varying prevalence of co-morbid diseases such as hypertension [33-35]. In addition, the prevalence of CKD among different racial and ethnic groups may vary according to the stage of CKD [36]. Thus, it may be necessary to perform and pool data from large, population-based studies to further delineate the contribution of individual CKD risk factors [37,38], allowing primary providers to screen for CKD akin to cardiovascular risk assessment based on the Framingham Study [39].

The use of mathematical formulas to estimate GFR rather than a timed urine collection for creatinine clearance is recommended for most patients [40], and the examination of a random urine sample for albumin and creatinine is the preferred screening method for albuminuria in adults [7,27]. However, despite this, more than half the physicians preferred a timed urine collection for creatinine, and in a hypothetical patient with diabetes and stage 3 CKD, only 70% of physicians elected to screen for microalbuminuria. Moreover, among those who did screen for microalbuminuria, only about a third would concomitantly obtain a urine creatinine concentration to normalize the albumin result (family medicine trainees: 19%, internal medicine trainees: 37%), as recommended by both the NKF and ADA [27,41]. Thus, further emphasis may need to be placed on hands-on implementation of clinical practice guidelines to improve the detection of subjects with CKD.

The vast majority of respondents identified blood pressure goal and first line antihypertensive agents for patients with CKD as recommended by the Seventh Report of the Joint National Committee on Prevention, Detection, Evaluation, and Treatment of High Blood Pressure [42]. However, practitioners, and in particular family medicine residents, were hesitant to use ACEi in the setting of stage 3 or 4 CKD. Data supporting the use of ACEi and ARB show that these agents are not only beneficial in patients with an abnormal serum creatinine, but also safe, as long as patients are carefully monitored and counseled [43,44]. Thus, it may be necessary to not only disseminate information about the usefulness of a given intervention, but also hands-on information about monitoring, prevention, and treatment of potential complications.

Almost all physicians recognized worsening hypertension, anemia, hyperkalemia, volume overload, and metabolic acidosis as complications of CKD. However, secondary hyperparathyroidism, malnutrition, and lipid disorders were identified by a significantly smaller proportion of respondents. Moreover, even though about 90% of physicians recognized anemia as a complication of CKD, only about 40% indicated that they would obtain a CBC in a hypothetical patient with diabetes and an eGFR of 34 ml/min/1.73 m^2^. Similarly, even among those who identified secondary hyperparathyroidism as a CKD complication, only a small fraction would order the appropriate screening test. This reflects the observation that the majority of patients with stage 4 CKD referred to a nephrologist were never screened for secondary hyperparathyroidism and had lower hemoglobin concentrations than those seen in a dedicated CKD clinic for at least six months [45]. In addition, the management of anemia and secondary hyperparathyroidism requires complex decision-making. These disorders have not only to be recognized as a complication of CKD, but in order to avoid future complications, screening needs to be initiated in stage 3 CKD and physicians also need to know about recommended treatment targets in order to either initiate therapy or consult an experienced specialist [9,12]. The data from this survey show that no more than 10% of physicians had the skills to integrate data from all three areas correctly, allowing them to formulate appropriate management plans for CKD patients with anemia or secondary hyperparathyroidism. Thus, unless an educational effort aimed specifically at non-nephrologists fills these knowledge gaps, nephrologists will likely have to co-manage patients with stage 3 and 4 CKD.

Clinical practice guidelines may present treatment goals, but often lack the necessary guidance as to why and how to reach these goals. Recently, an approach to link evidence with practice in CKD care, which may be particularly useful in managed care settings, has been published [46]; however, none of the strategies to improve compliance tested thus far have proven fail-safe [47]. Deficits in compliance with clinical practice guidelines are not unique to nephrology, and the reasons for non-compliance are complex [48]. Recent data show that there is an acute lack of awareness of clinical practice guidelines for CKD patients among primary providers [22,49]. Based on the data presented here, educational efforts aimed at improving CKD care will have to start during the training program, even though this may be a challenging undertaking given the requirements and restrictions placed upon US training programs [50]. Specifically, trainees will need to be instructed about prevalence of CKD, risk factors for CKD, screening methods for CKD and its complications, and treatment options to slow the progression of CKD and reduce morbidity and mortality [51-54]. In order to succeed, guidelines will have to be presented in a manner that fosters reflection, critically appraises clinical evidence, is practice-based, and is easy to understand for practitioners and patients alike [48,55].

This study has several important limitations. Given that it is based on a voluntary, anonymous online survey, certain important variables, such as training program size and setting, or the presence or absence of formal teaching in CKD care, cannot be controlled. In addition, demographic characteristics of the study cohort cannot be verified. Finally we cannot be certain that the answers truly reflect the knowledge of the person completing the survey, given that the survey instrument has not been formally validated in this respect, although we did include nephrologists who might serve as a positive control group (see Additional file 2). However, given that we received a large number of responses from across the United States, we believe that the data presented here are representative and covey important information about practice patterns of future providers.

## Conclusion

In conclusion, the results from this survey suggest that physicians currently in training have significant knowledge gaps in CKD management, and with few exceptions attendings' responses closely mimicked those of trainees. These finding suggests that education is needed to raise awareness of clinical practice guidelines and recommendations for patients with CKD among future practitioners. Surveys similar to ours may be useful in the planning of training programs to assess areas that need additional emphasis.

## Competing interests

Oliver Lenz has received educational grants, honoraria, and consulting fees from Abbott Laboratories, Amgen, Genzyme, OrthoBiotech, and Pfizer.

## Authors' contributions

Oliver Lenz designed, published, and analyzed the survey and wrote the manuscript. Alessia Fornoni assisted in the design of the survey and co-wrote the manuscript.

## Pre-publication history

The pre-publication history for this paper can be accessed here:



## Supplementary Material

Additional file 2 The survey instrument was administered to ten nephrology fellows and attending physicians at the investigators' center. Their answers served as a reference as to what to expect from a (presumably) knowledgeable population. All nephrology fellows had completed formal theoretical and practical training in CKD care. Click here for file.

Additional File 1 The survey instrument was administered as shown except that a random sequence was chosen for the options in items 7, 8, 10, 11, 12, 17 and 18 whenever a survey was begun. Click here for file
