# Supplementary material for: Chronic kidney disease care delivered by US family medicine and internal medicine trainees: results from an online survey
Source: BMC Med. 2006 Dec 12;4:30. doi: 10.1186/1741-7015-4-30 (PMC1713248; doi:10.1186/1741-7015-4-30)
Supplement: Additional file 2 — The survey instrument was administered to ten nephrology fellows and attending physicians at the investigators' center. Their answers served as a reference as to what to expect from a (presumably) knowledgeable population. All nephrology fellows had completed formal theoretical and practical training in CKD care. [file 1741-7015-4-30-S2.pdf]

Results Summary

Show All Pages and Questions

Export... | View Detail >>

Filter Results

To analyze a subset of your data, you can create one or more filters.

Add Filter... **Total:** 10

Visible: 10

Share Results

Your results can be shared with others, without giving access to your account.

Configure... **Status:** Enabled

Reports: Summary and Detail

2. page 2

1. Professional Title

	Response Percent	Response Total
Medical Student	0%	0
**Physician**	**100%**	**10**
Nurse Practitioner	0%	0
Physician Assistant	0%	0
Other (please specify)	0%	0
	**Total Respondents**	**10**
	**(skipped this question)**	**0**

3. Subspecialty

1. Subspecialty (choose field of major activity)

	Response Percent	Response Total
Cardiology	0%	0
Clinical Pharmacology	0%	0
Endocrinology/Diabetes/Metabolism	0%	0
Gastroenterology	0%	0
General Internal Medicine	0%	0
Gerontology & Geriatric Medicine	0%	0
Hematology/Oncology	0%	0
Infectious Diseases	0%	0
Laboratory Medicine	0%	0
**Nephrology & Hypertension**	**100%**	**8**
Pulmonary & Critical Care	0%	0
Rheumatology & Immunology	0%	0
Other (please specify)	0%	0

**Total Respondents**		**8**
(skipped this question)		2

4. Specialty

1. Primary Specialty

	Response Percent	Response Total
Anesthesiology	0%	0
Dermatology	0%	0
Family Medicine	0%	0
**Internal Medicine & Subspecialties**	**80%**	**8**
Neurological Surgery	0%	0
Neurology	0%	0
Obstetrics & Gynecology	0%	0
Ophthalmology	0%	0
Orthopaedics and Rehabilitation	0%	0
Otolaryngology	0%	0
Pathology	0%	0
Pediatrics	0%	0
Physical Therapy	0%	0
Psychiatry	0%	0
Radiation Oncology	0%	0
Radiology	0%	0
Rehabilitaion Medicine	0%	0
Surgery	0%	0
View Other (please specify)	20%	2
**Total Respondents**		**10**
(skipped this question)		0

5. Practice type

1. Practice type

	Response Percent	Response Total
Not seeing patients	0%	0
**Academic**	**100%**	**10**
Non-academic solo practice	0%	0
Non-academic single-specialty group practice	0%	0
Non-academic multi-specialty group practice	0%	0
**Total Respondents**		**10**
(skipped this question)		0

2. ZIP code of primary practice site (choose only one):

	Response Percent	Response Total
99999 - no practice	10%	1
**33136 - JMH, VAMC, UMHC/Sylvester**	**90%**	**9**
Other (please specify)	0%	0
**Total Respondents**		**10**
(skipped this question)		0

6. Survey

1. How many patients do you see per week?

Use an average if you are not seeing patients every week.

	Response Percent	Response Total
less than 15	20%	2
15-25	20%	2
**26-50**	**30%**	**3**
51-100	20%	2
101-150	10%	1
more than 150	0%	0
**Total Respondents**		**10**
(skipped this question)		0

2. How many patients in your practice have any degree of kidney failure?

Include patients with normal creatinine clearance and evidence of kidney damage such as microalbuminuria, proteinuria, or hematuria.

	Response Percent	Response Total
none	0%	0
1-10%	20%	2
11-30%	0%	0
31-50%	10%	1
51-70%	0%	0
**>70%**	**70%**	**7**
**Total Respondents**		**10**
(skipped this question)		0

7. Survey2

1. In your opinion, what is the best measure of kidney function?

Choose only one answer.

	Response Percent	Response Total

	Response Percent	Response Total
24-hour urine collection for creatinine clearance	20%	2
Serum creatinine	0%	0
**Estimated glomerular filtration rate (GFR) using a mathematical formula**	**70%**	**7**
24-hour urine collection for urea clearance	10%	1
BUN	0%	0
Renal ultrasound	0%	0
Renogram with lasix	0%	0
	**Total Respondents**	**10**
	**(skipped this question)**	**0**

8. Survey3

1. In your opinion, how do the following factors influence the risk for developing chronic kidney disease?

	No influence	Minimally increased	Highly increased	Response Average
Diabetes	0% (0)	0% (0)	**100% (10)**	**3.00**
Hypertension	0% (0)	0% (0)	**100% (10)**	**3.00**
Autoimmune diseases	0% (0)	**50% (5)**	**50% (5)**	**2.50**
Recurrent urinary tract infections	0% (0)	**90% (9)**	10% (1)	**2.10**
Kidney stones	0% (0)	**80% (8)**	20% (2)	**2.20**
Lower urinary tract obstruction	20% (2)	**40% (4)**	**40% (4)**	**2.20**
Family history of kidney failure	0% (0)	**60% (6)**	40% (4)	**2.40**
Recovery from acute renal failure	0% (0)	**80% (8)**	20% (2)	**2.20**
Age above 65 years	0% (0)	**50% (5)**	**50% (5)**	**2.50**
African American race	0% (0)	30% (3)	**70% (7)**	**2.70**
Hispanic ethnicity	20% (2)	**50% (5)**	30% (3)	**2.10**
Body mass index between 18 and 22	**60% (6)**	20% (2)	20% (2)	**1.60**
Cigarette smoking	0% (0)	**60% (6)**	40% (4)	**2.40**
Chronic heart failure	0% (0)	40% (4)	**60% (6)**	**2.60**
			**Total Respondents**	**10**
			**(skipped this question)**	**0**

9. Survey4

1. What is your blood pressure goal for a patient with a creatinine clearance of 40 ml/min?

	Response Percent	Response Total
less than 140/90	0%	0
less than 130/90	10%	1
**less than 130/80**	**70%**	**7**
less than 125/75	20%	2

less than 120/70	0%	0
I don't know	0%	0
**Total Respondents**		**10**
**(skipped this question)**		**0**

10. Survey4b

1. In a patient with mild to moderate chronic kidney disase, which of the following do you consider first-line antihypertensive agents to slow the progression of kidney disease?

Check all that apply.

	Response Percent	Response Total
Beta blockers	0%	0
Loop diuretics	0%	0
Thiazide diuretics	20%	2
**Angiotensin converting enzyme inhibitors**	**90%**	**9**
Angiotensin receptor blockers	70%	7
Dihidropyridine calcium channel blockers	0%	0
Non-dihidropyridine calcium channel blockers	0%	0
Alpha blockers	0%	0
Other (please specify)	0%	0
**Total Respondents**		**10**
**(skipped this question)**		**0**

11. Survey6

1. Your see a 55-year old African American woman with type 2 diabetes and hypertension. Her medications are insulin and a thiazide diuretic. Her blood pressure is well controlled, and she has no complaints. Her serum creatinine has increased to 2 mg/dl over the past two years. Which of the following laboratory tests would you order in a patient like this?

	Response Percent	Response Total
Serum calcium	40%	4
Serum phosphate	50%	5
Intact PTH	70%	7
CBC	60%	6
**Glycohemoglobin (HbA1c)**	**90%**	**9**
Lipid panel	80%	8
**Urine analysis**	**90%**	**9**
24-hour urine collection for creatinine	0%	0
24-hour urine collection for protein	40%	4
Random (spot) urine sample for microalbumin	80%	8
Random (spot) urine sample for creatinine	50%	5
Random (spot) urine sample for		

electrolytes	0%	0
Serum albumin	40%	4
Liver function panel	20%	2
	**Total Respondents**	**10**
	**(skipped this question)**	**0**

12. Survey5

1. Which of the following conditions may be a consequence of chronic kidney disease? Check all that apply.

	Response Percent	Response Total
**Anemia**	**100%**	**10**
**Metabolic acidosis**	**100%**	**10**
Respiratory acidosis	10%	1
**Secondary hyperparathyroidism**	**100%**	**10**
**Worsening hypertension**	**100%**	**10**
Malnutrition	90%	9
Obesity	10%	1
Dyslipidemia	80%	8
**Volume overload**	**100%**	**10**
Hirsutism	10%	1
**Increased serum potassium**	**100%**	**10**
	**Total Respondents**	**10**
	**(skipped this question)**	**0**

13. Survey 6aa

1. At what level of creatinine clearance (or glomerular filtration rate) do you currently screen your patient for each of the following conditions?

	below 90 ml/min	below 60 ml/min	below 30 ml/min	below 15 ml/min	I do not screen	Response Average
Anemia	20% (2)	**60% (6)**	20% (2)	0% (0)	0% (0)	**2.00**
Metabolic acidosis	0% (0)	40% (4)	**60% (6)**	0% (0)	0% (0)	**2.60**
Secondary hyperparathyroidism	10% (1)	**80% (8)**	0% (0)	10% (1)	0% (0)	**2.10**
Malnutrition	0% (0)	**50% (5)**	**50% (5)**	0% (0)	0% (0)	**2.50**
					**Total Respondents**	**10**
					**(skipped this question)**	**0**

14. Survey 6b

1. At what hemoglobin concentration would you intervene (start treatment or request a consult) in a patient with a creatinine clearance of 40 ml/min and anemia of chronic disease?

Response Percent	Response Total

	Response Percent	Response Total
Hgb below 8 g/dl	0%	0
Hgb below 9 g/dl	0%	0
Hgb below 10 g/dl	30%	3
**Hgb below 11 g/dl**	**40%**	**4**
Hgb below 12 g/dl	30%	3
Hgb below 13 g/dl	0%	0
**Total Respondents**		**10**
**(skipped this question)**		**0**

15. Survey 6ba

1. At what intact PTH concentration would you intervene (start treatment or request a consult) in a patient with a creatinine clearance of 40 ml/min and secondary hyperparathyroidism?

	Response Percent	Response Total
intact PTH above 30 pg/ml	0%	0
intact PTH above 50 pg/ml	0%	0
intact PTH above 70 pg/ml	40%	4
**intact PTH above 110 pg/ml**	**50%**	**5**
intact PTH above 150 pg/ml	0%	0
I do not know	10%	1
**Total Respondents**		**10**
**(skipped this question)**		**0**

16. Survey 6c

1. What medications, if any, would you use to treat this patient's anemia (check all that apply)?

	Response Percent	Response Total
Oral iron	50%	5
Intravenous iron	70%	7
**Erythropoietin or darbapoietin**	**90%**	**9**
No medications	0%	0
View Other (please specify)	10%	1
**Total Respondents**		**10**
**(skipped this question)**		**0**

2. What consultations, if any, would you request?

	Response Percent	Response Total
**Nephrology consult**	**70%**	**7**
Hematology consult	0%	0
No consultation	30%	3
Other (please specify)	0%	0

**Total Respondents**	**10**
(skipped this question)	0

17. Survey 6d

1. What medications, if any, would you use to treat this patient's elevated PTH?

	Response Percent	Response Total
Calcium supplementation	0%	0
Phosphate binder	0%	0
**1,25-Vitamin D (calcitriol, paricalcitol, or doxercalciferol)**	**80%**	**8**
25-Vitamin D (ergocalciferol)	0%	0
No Medication	20%	2
Other (please specify)	0%	0
**Total Respondents**		**10**
(skipped this question)		0

2. What consultations, if any, would you request?

	Response Percent	Response Total
**Nephrology consultation**	**70%**	**7**
Endocrinology consultation	0%	0
No consultation	30%	3
Other (please specify)	0%	0
**Total Respondents**		**10**
(skipped this question)		0

18. Survey7

1. In your opinion, if a patient with a creatinine clearance of 40 ml/min develops secondary hyperparathyroidism, what combination of serum calcium, serum phosphate, and PTH concentrations is most likely?

	Response Percent	Response Total
**Low calcium, high phosphate, high PTH**	**50%**	**5**
High calcium, low phosphate, high PTH	0%	0
Low calcium, normal phosphate, high PTH	0%	0
**Normal calcium, normal phosphate, high PTH**	**50%**	**5**
Low calcium, high phosphate, normal PTH	0%	0
Other (please specify)	0%	0
**Total Respondents**		**10**
(skipped this question)		0

19. Survey8

1. At what level of kidney function would you refer a patient with diabetic nephropathy to a nephrologist?

	Response Percent	Response Total
As soon as the serum creatinine is abnormal	0%	0
GFR or CrCl less than 90 ml/min	20%	2
**GFR or CrCl less than 60 ml/min**	**40%**	**4**
GFR or CrCl less than 30 ml/min	0%	0
GFR or CrCl less than 15 ml/min	0%	0
**as soon as microalbuminuria is present, regardless of creatinine clearance**	**40%**	**4**
	**Total Respondents**	**10**
	**(skipped this question)**	**0**

20. Survey9

1. Your patient has chronic kidney disease and wants to start hemodialysis when the kidneys fail. In your opinion, at what level of glomerular filtration rate (GFR) or creatinine clearance (CrCl) should a vascular access (fistula) be placed?

	Response Percent	Response Total
GFR or CrCl less than 50 ml/min	0%	0
**GFR or CrCl less than 25 ml/min**	**100%**	**10**
GFR or CrCl less than 15 ml/min	0%	0
After patient started dialysis	0%	0
I am not sure	0%	0
	**Total Respondents**	**10**
	**(skipped this question)**	**0**

21. Survey10

1. Your patient is a 37-year old Non-Hispanic Caucasian man with hypertension, microalbuminuria (40 mg/day), and a blood pressure of 145/95 mmHg. He is taking a thiazide diuretic. Would you add an angiotensin converting enzyme inhibitor if the patient's serum creatinine concentrations fell within one of the following ranges?

	Yes	No	Response Average
0.6-1.4 mg/dl	**100% (10)**	0% (0)	**1.00**
1.5-2.0 mg/dl	**100% (10)**	0% (0)	**1.00**
2.1-2.5 mg/dl	**100% (10)**	0% (0)	**1.00**
2.6-3.0 mg/dl	**100% (10)**	0% (0)	**1.00**
3.1-4.0 mg/dl	**100% (10)**	0% (0)	**1.00**
above 4.0 mg/dl	**80% (8)**	20% (2)	**1.20**
		**Total Respondents**	**10**
		**(skipped this question)**	**0**

22. Survey11

1. Your patient is a 37-year old African American man with hypertension, microalbuminuria (40 mg/day), and a blood pressure of 145/95 mmHg. He is taking a thiazide diuretic. Would you add an angiotensin converting enzyme inhibitor if the patient's serum creatinine concentrations fell within one of the following ranges?

	Yes	No	Response Average
0.6-1.4 mg/dl	**100% (10)**	0% (0)	**1.00**
1.5-2.0 mg/dl	**100% (10)**	0% (0)	**1.00**
2.1-2.5 mg/dl	**100% (10)**	0% (0)	**1.00**
2.6-3.0 mg/dl	**100% (10)**	0% (0)	**1.00**
3.1-4.0 mg/dl	**100% (10)**	0% (0)	**1.00**
above 4.0 mg/dl	**90% (9)**	10% (1)	**1.10**
		**Total Respondents**	**10**
		**(skipped this question)**	**0**

23. All of your answers are recorded anonymously.

1. Your age (not required)

View **Total Respondents**	**1**
**(skipped this question)**	**9**

2. Your gender (not required)

	Response Percent	Response Total
Female	20%	1
**Male**	**80%**	**4**
**Total Respondents**		**5**
**(skipped this question)**		**5**

3. Level of training

	Response Percent	Response Total
Student	0%	0
Intern	0%	0
Resident	0%	0
**Fellow**	**60%**	**6**
Attending	40%	4
**Total Respondents**		**10**
**(skipped this question)**		**0**

4. Years in practice

Response Percent	Response Total

**0-3**		**50%**	**5**
4-6		10%	1
7-9		10%	1
10+		30%	3
		**Total Respondents**	**10**
		**(skipped this question)**	**0**

24. Thank you for participating in this survey. Please give us your feedback.

1. Please rate this survey

	Strongly disagree	Somewhat disagree	Neutral	Somewhat agree	Strongly agree	Response Average
Questions were clear	10% (1)	0% (0)	10% (1)	30% (3)	**50% (5)**	**4.10**
Questions were relevant	10% (1)	0% (0)	10% (1)	30% (3)	**50% (5)**	**4.10**
Length of survey was acceptable	0% (0)	10% (1)	10% (1)	**40% (4)**	**40% (4)**	**4.10**
					**Total Respondents**	**10**
					**(skipped this question)**	**0**

2. Comments:

View **Total Respondents**	**1**
**(skipped this question)**	**9**

SurveyMonkey is Hiring! | Privacy Statement | Contact Us | Logout

Copyright ©1999-2006 SurveyMonkey.com. All Rights Reserved.
No portion of this site may be copied without the express written consent of SurveyMonkey.com.
