# Supplementary material for: Chronic kidney disease care delivered by US family medicine and internal medicine trainees: results from an online survey
Source: BMC Med. 2006 Dec 12;4:30. doi: 10.1186/1741-7015-4-30 (PMC1713248; doi:10.1186/1741-7015-4-30)
Supplement: Additional File 1 — The survey instrument was administered as shown except that a random sequence was chosen for the options in items 7, 8, 10, 11, 12, 17 and 18 whenever a survey was begun. [file 1741-7015-4-30-S1.pdf]

Design Survey

Show All Pages and Questions

<< Back | Preview

To change the **look** of your survey, select a choice below. Click 'Add' to create your own custom theme.

Theme: Nephrology | Add | Edit | Delete

Division of Nephrology - Chronic Kidney Disease Survey

Edit Title | Edit Numbering | Edit Logo

Add Page

1. Begin the survey.

Edit Page | Delete Page | Copy/Move | Add Logic

Add Question | Add Page

Edit | Delete | Copy/Move

This survey is designed to detect practice patterns; it does not seek right or wrong answers. Please answer all questions according to what you do in your everyday clinical practice.

All answers are recorded in an anonymous fashion.

Please click on "next" to start the survey.

Add Question | Add Page

2. page 2

Edit Page | Delete Page | Copy/Move | Edit Logic

Progress: ■□□□□□□□□□□□

Add Question | Add Page

Edit | Delete | Copy/Move | Add Logic

*** Professional Title**

- Medical Student
- Physician
- Nurse Practitioner
- Physician Assistant
- Other (please specify)

Add Question | Add Page

3. Subspecialty

Edit Page | Delete Page | Copy/Move | Edit Logic

Progress: ■□□□□□□□□□□□

Add Question | Add Page

Edit | Delete | Copy/Move | Add Logic

*** Subspecialty (choose field of major activity)**

- Cardiology
- Clinical Pharmacology
- Endocrinology/Diabetes/Metabolism
- Gastroenterology
- General Internal Medicine
- Gerontology & Geriatric Medicine
- Hematology/Oncology
- Infectious Diseases
- Laboratory Medicine
- Nephrology & Hypertension
- Pulmonary & Critical Care
- Rheumatology & Immunology
- Other (please specify)

Add Question | Add Page

4. Specialty

Edit Page | Delete Page | Copy/Move | Add Logic

Progress: ■□□□□□□□□□□□

Add Question | Add Page

Edit | Delete | Copy/Move | Edit Logic

*** Primary Specialty**

- Anesthesiology
- Dermatology
- Family Medicine
- Internal Medicine & Subspecialties
- Neurological Surgery
- Neurology
- Obstetrics & Gynecology
- Ophthalmology
- Orthopaedics and Rehabilitation
- Otolaryngology
- Pathology
- Pediatrics
- Physical Therapy
- Psychiatry
- Radiation Oncology
- Radiology
- Rehabilitaion Medicine
- Surgery
- Other (please specify)

Add Question | Add Page

5. Practice type

Edit Page | Delete Page | Copy/Move | Add Logic

Progress: ■■□□□□□□□□□□

Add Question | Add Page

Edit | Delete | Copy/Move | Add Logic

*** Practice type**

- Not seeing patients
- Academic
- Non-academic solo practice
- Non-academic single-specialty group practice
- Non-academic multi-specialty group practice

Add Question | Add Page

Edit | Delete | Copy/Move | Add Logic

*** ZIP code of primary practice site (choose only one):**

- 99999 - no practice
- 33136 - JMH, VAMC, UMHC/Sylvester
- Other (please specify)

Add Question | Add Page

6. Survey

Edit Page | Delete Page | Copy/Move | Add Logic

Progress: ■■□□□□□□□□□□

Add Question | Add Page

Edit | Delete | Copy/Move | Add Logic

*** How many patients do you see per week?**

Use an average if you are not seeing patients every week.

- less than 15
- 15-25
- 26-50
- 51-100
- 101-150
- more than 150

Add Question | Add Page

Edit | Delete | Copy/Move | Add Logic

*** How many patients in your practice have any degree of kidney failure?**

Include patients with normal creatinine clearance and evidence of kidney damage such as microalbuminuria, proteinuria, or hematuria.

- none
- 1-10%
- 11-30%
- 31-50%

51-70%
>70%

Add Question Add Page

7. Survey2 Edit Page Delete Page Copy/Move Add Logic

Progress: ■■■□□□□□□□□□□

Add Question Add Page

Edit Delete Copy/Move Add Logic

*** In your opinion, what is the best measure of kidney function?**

Choose only one answer.

- 24-hour urine collection for creatinine clearance
- Renogram with lasix
- Renal ultrasound
- 24-hour urine collection for urea clearance
- BUN
- Estimated glomerular filtration rate (GFR) using a mathematical formula
- Serum creatinine

Add Question Add Page

8. Survey3 Edit Page Delete Page Copy/Move Add Logic

Progress: ■■■□□□□□□□□□□

Add Question Add Page

Edit Delete Copy/Move

*** In your opinion, how do the following factors influence the risk for developing chronic kidney disease?**

	No influence	Minimally increased	Highly increased
Cigarette smoking			
Recurrent urinary tract infections			
Age above 65 years			
Kidney stones			
Lower urinary tract obstruction			
Recovery from acute renal failure			
Chronic heart failure			
Body mass index between 18 and 22			
African American race			
Hispanic ethnicity			
Family history of kidney failure			
Autoimmune diseases			

Hypertension			
Diabetes			

Add Question Add Page

9. Survey4

Edit Page Delete Page Copy/Move Add Logic

Progress: ■■■■□□□□□□□□□

Add Question Add Page

Edit Delete Copy/Move Add Logic

*** What is your blood pressure goal for a patient with a creatinine clearance of 40 ml/min?**

- less than 140/90
- less than 130/90
- less than 130/80
- less than 125/75
- less than 120/70
- I don't know

Add Question Add Page

10. Survey4b

Edit Page Delete Page Copy/Move Add Logic

Progress: ■■■■□□□□□□□□□

Add Question Add Page

Edit Delete Copy/Move Add Logic

*** In a patient with mild to moderate chronic kidney disase, which of the following do you consider first-line antihypertensive agents to slow the progression of kidney disease?**

Check all that apply.

- Alpha blockers
- Non-dihidropyridine calcium channel blockers
- Angiotensin receptor blockers
- Angiotensin converting enzyme inhibitors
- Thiazide diuretics
- Loop diuretics
- Dihidropyridine calcium channel blockers
- Beta blockers
- Other (please specify)

Add Question Add Page

11. Survey6

Edit Page Delete Page Copy/Move Add Logic

Progress: ■■■■■□□□□□□□□

Add Question Add Page

Edit | Delete | Copy/Move | Add Logic

*** Your see a 55-year old African American woman with type 2 diabetes and hypertension. Her medications are insulin and a thiazide diuretic. Her blood pressure is well controlled, and she has no complaints. Her serum creatinine has increased to 2 mg/dl over the past two years.**

Which of the following laboratory tests would you order in a patient like this?

- [ ] Lipid panel
- [ ] Random (spot) urine sample for microalbumin
- [ ] Random (spot) urine sample for creatinine
- [ ] CBC
- [ ] Liver function panel
- [ ] Serum calcium
- [ ] Urine analysis
- [ ] Serum phosphate
- [ ] Intact PTH
- [ ] Glycohemoglobin (HbA1c)
- [ ] Serum albumin
- [ ] Random (spot) urine sample for electrolytes
- [ ] 24-hour urine collection for protein
- [ ] 24-hour urine collection for creatinine

Add Question | Add Page

12. Survey5

Edit Page | Delete Page | Copy/Move | Add Logic

Progress: ■■■■■□□□□□□□

Add Question | Add Page

Edit | Delete | Copy/Move | Add Logic

*** Which of the following conditions may be a consequence of chronic kidney disease?**

Check all that apply.

- [ ] Anemia
- [ ] Metabolic acidosis
- [ ] Respiratory acidosis
- [ ] Secondary hyperparathyroidism
- [ ] Worsening hypertension
- [ ] Increased serum potassium
- [ ] Dyslipidemia
- [ ] Malnutrition
- [ ] Volume overload
- [ ] Hirsutism
- [ ] Obesity

Add Question | Add Page

13. Survey 6aa

Edit Page | Delete Page | Copy/Move | Add Logic

Progress: ■■■■■■□□□□□□□

Add Question Add Page

Edit Delete Copy/Move

*** At what level of creatinine clearance (or glomerular filtration rate) do you currently screen your patient for each of the following conditions?**

	below 90 ml/min	below 60 ml/min	below 30 ml/min	below 15 ml/min	I do not screen
Metabolic acidosis	○	○	○	○	○
Anemia	○	○	○	○	○
Malnutrition	○	○	○	○	○
Secondary hyperparathyroidism	○	○	○	○	○

Add Question Add Page

14. Survey 6b

Edit Page Delete Page Copy/Move Add Logic

Progress: ■■■■■■□□□□□□□

Add Question Add Page

Edit Delete Copy/Move Add Logic

*** At what hemoglobin concentration would you intervene (start treatment or request a consult) in a patient with a creatinine clearance of 40 ml/min and anemia of chronic disease?**

- Hgb below 8 g/dl
- Hgb below 9 g/dl
- Hgb below 10 g/dl
- Hgb below 11 g/dl
- Hgb below 12 g/dl
- Hgb below 13 g/dl

Add Question Add Page

15. Survey 6ba

Edit Page Delete Page Copy/Move Add Logic

Progress: ■■■■■■■□□□□□□

Add Question Add Page

Edit Delete Copy/Move Add Logic

*** At what intact PTH concentration would you intervene (start treatment or request a consult) in a patient with a creatinine clearance of 40 ml/min and secondary hyperparathyroidism?**

- intact PTH above 30 pg/ml
- intact PTH above 50 pg/ml
- intact PTH above 70 pg/ml
- intact PTH above 110 pg/ml
- intact PTH above 150 pg/ml
-

I do not know

Add Question Add Page

16. Survey 6c

Edit Page Delete Page Copy/Move Add Logic

Progress: ■■■■■■■□□□□□□

Add Question Add Page

Edit Delete Copy/Move

Your patient is a 40-year old woman with hypertension. She has the following laboratory data:

Creatinine clearance	40 ml/min	(normal:90-140)
Hgb:	8.0 g/dl	(normal:12-16)
Iron:	40 mcg/dl	(normal:65-165)
TIBC:	400 mcg/dl	(normal:300-450)
Ferritin:	40 ng/ml	(normal:20-300)

Add Question Add Page

Edit Delete Copy/Move Add Logic

*** What medications, if any, would you use to treat this patient's anemia (check all that apply)?**

- Oral iron
- Intravenous iron
- Erythropoietin or darbapoietin
- No medications
- Other (please specify)

Add Question Add Page

Edit Delete Copy/Move Add Logic

*** What consultations, if any, would you request?**

- Nephrology consult
- Hematology consult
- No consultation
- Other (please specify)

Add Question Add Page

17. Survey 6d

Edit Page Delete Page Copy/Move Add Logic

Progress: ■■■■■■■■□□□□□

Add Question Add Page

Edit Delete Copy/Move

Your patient is a 40-year old woman with hypertension. She has the following laboratory

data:

```
Creatinine clearance      40 ml/min   (normal:90-140)
Calcium:                 8.5 mg/dl    (normal:8.5-10.5)
Phosphate:               2.9 mg/dl    (normal:2.0-4.3)
PTH (intact):            120 pg/ml    (normal:10-55)
25-OH Vitamin D:          40 ng/ml    (normal:20-56)
```

Add Question | Add Page

Edit | Delete | Copy/Move | Add Logic

*** What medications, if any, would you use to treat this patient's elevated PTH?**

- [ ] Calcium supplementation
- [ ] Phosphate binder
- [ ] 1,25-Vitamin D (calcitriol, paricalcitol, or doxercalciferol)
- [ ] 25-Vitamin D (ergocalciferol)
- [ ] No Medication
- [ ] Other (please specify)

Add Question | Add Page

Edit | Delete | Copy/Move | Add Logic

*** What consultations, if any, would you request?**

- [ ] Nephrology consultation
- [ ] Endocrinology consultation
- [ ] No consultation
- [ ] Other (please specify)

Add Question | Add Page

18. Survey7

Edit Page | Delete Page | Copy/Move | Add Logic

Progress: ■■■■■■■■■■□□□

Add Question | Add Page

Edit | Delete | Copy/Move | Add Logic

*** In your opinion, if a patient with a creatinine clearance of 40 ml/min develops secondary hyperparathyroidism, what combination of serum calcium, serum phosphate, and PTH concentrations is most likely?**

- ○ Low calcium, normal phosphate, high PTH
- ○ High calcium, low phosphate, high PTH
- ○ Low calcium, high phosphate, high PTH
- ○ Low calcium, high phosphate, normal PTH
- ○ Normal calcium, normal phosphate, high PTH
- ○ Other (please specify)

Add Question | Add Page

19. Survey8

Edit Page | Delete Page | Copy/Move | Add Logic

Progress: ■■■■■■■■■■□□□

Add Question | Add Page

Edit | Delete | Copy/Move | Add Logic

*** At what level of kidney function would you refer a patient with diabetic nephropathy to a nephrologist?**

- As soon as the serum creatinine is abnormal
- GFR or CrCl less than 90 ml/min
- GFR or CrCl less than 60 ml/min
- GFR or CrCl less than 30 ml/min
- GFR or CrCl less than 15 ml/min
- as soon as microalbuminuria is present, regardless of creatinine clearance

Add Question | Add Page

20. Survey9

Edit Page | Delete Page | Copy/Move | Add Logic

Progress: ■■■■■■■■■■■□□

Add Question | Add Page

Edit | Delete | Copy/Move | Add Logic

*** Your patient has chronic kidney disease and wants to start hemodialysis when the kidneys fail. In your opinion, at what level of glomerular filtration rate (GFR) or creatinine clearance (CrCl) should a vascular access (fistula) be placed?**

- GFR or CrCl less than 50 ml/min
- GFR or CrCl less than 25 ml/min
- GFR or CrCl less than 15 ml/min
- After patient started dialysis
- I am not sure

Add Question | Add Page

21. Survey10

Edit Page | Delete Page | Copy/Move | Add Logic

Progress: ■■■■■■■■■■■■□

Add Question | Add Page

Edit | Delete | Copy/Move

*** Your patient is a 37-year old Non-Hispanic Caucasian man with hypertension, microalbuminuria (40 mg/day), and a blood pressure of 145/95 mmHg. He is taking a thiazide diuretic. Would you add an angiotensin converting enzyme inhibitor if the patient's serum creatinine concentrations fell within one of the following ranges?**

	Yes	No
0.6-1.4 mg/dl	○	○
1.5-2.0 mg/dl	○	○
2.1-2.5 mg/dl	○	○
2.6-3.0 mg/dl	○	○

3.1-4.0 mg/dl	◯	◯
above 4.0 mg/dl	◯	◯

Add Question | Add Page

22. Survey11

Edit Page | Delete Page | Copy/Move | Add Logic

Progress: ■■■■■■■■■■■■□

Add Question | Add Page

Edit | Delete | Copy/Move

*** Your patient is a 37-year old African American man with hypertension, microalbuminuria (40 mg/day), and a blood pressure of 145/95 mmHg. He is taking a thiazide diuretic. Would you add an angiotensin converting enzyme inhibitor if the patient's serum creatinine concentrations fell within one of the following ranges?**

	Yes	No
0.6-1.4 mg/dl	◯	◯
1.5-2.0 mg/dl	◯	◯
2.1-2.5 mg/dl	◯	◯
2.6-3.0 mg/dl	◯	◯
3.1-4.0 mg/dl	◯	◯
above 4.0 mg/dl	◯	◯

Add Question | Add Page

23. All of your answers are recorded anonymously.

Edit Page | Delete Page | Copy/Move | Add Logic

Progress: ■■■■■■■■■■■■■

Add Question | Add Page

Edit | Delete | Copy/Move

Your age (not required)

Add Question | Add Page

Edit | Delete | Copy/Move | Add Logic

Your gender (not required)

Female	Male
◯	◯

Add Question | Add Page

Edit | Delete | Copy/Move | Add Logic

*** Level of training**

Student	Intern	Resident	Fellow	Attending
◯	◯	◯	◯	◯

Add Question | Add Page

Edit | Delete | Copy/Move | Add Logic

* Years in practice

0-3	4-6	7-9	10+
○	○	○	○

Add Question | Add Page

24. Thank you for participating in this survey. Please give us your feedback.

Edit Page | Delete Page

Progress: ■■■■■■■■■■■■■

Add Question | Add Page

Edit | Delete | Copy/Move

Please rate this survey

	Strongly disagree	Somewhat disagree	Neutral	Somewhat agree	Strongly agree
Questions were clear	○	○	○	○	○
Questions were relevant	○	○	○	○	○
Length of survey was acceptable	○	○	○	○	○

Add Question | Add Page

Edit | Delete | Copy/Move

Comments:

Add Question | Add Page

25. The end

Edit Page | Delete Page | Copy/Move | Add Logic

Add Question | Add Page

Edit | Delete | Copy/Move

Thank you for participating in this survey.

For questions regarding this survey please contact:

Oliver Lenz, MD
Division of Nephrology & Hypertension
1600 NW 10th Ave Rm 7168
Miami, FL 33136

Add Question | Add Page

<< Back Preview

SurveyMonkey is Hiring! | Privacy Statement | Contact Us | Logout

Copyright ©1999-2006 SurveyMonkey.com. All Rights Reserved.
No portion of this site may be copied without the express written consent of SurveyMonkey.com.
